# Supplementary material for: Supramolecular Self-Assembly Strategy towards Fabricating Mesoporous Nitrogen-Rich Carbon for Efficient Electro-Fenton Degradation of Persistent Organic Pollutants
Source: Nanomaterials (Basel). 2022 Aug 17;12(16):2821. doi: 10.3390/nano12162821 (PMC9413581; doi:10.3390/nano12162821)
Supplement: Supplementary file 1 [file nanomaterials-12-02821-s001.zip › nanomaterials-1870082-supplementary.pdf]

Supplementary Materials

Supramolecular Self-Assembly Strategy towards Fabricating Mesoporous Nitrogen-Rich Carbon for Efficient Electro-Fenton Degradation of Persistent Organic Pollutants

Ye Chen †, Miao Tian *,† and Xupo Liu

School of Materials Science and Engineering, Henan Engineering Research Center of Design and Recycle for Advanced Electrochemical Energy Storage Materials, Henan Normal University, Xinxiang 453007, China

* Correspondence: tianmiao@htu.edu.cn

† These authors contributed equally to this work.

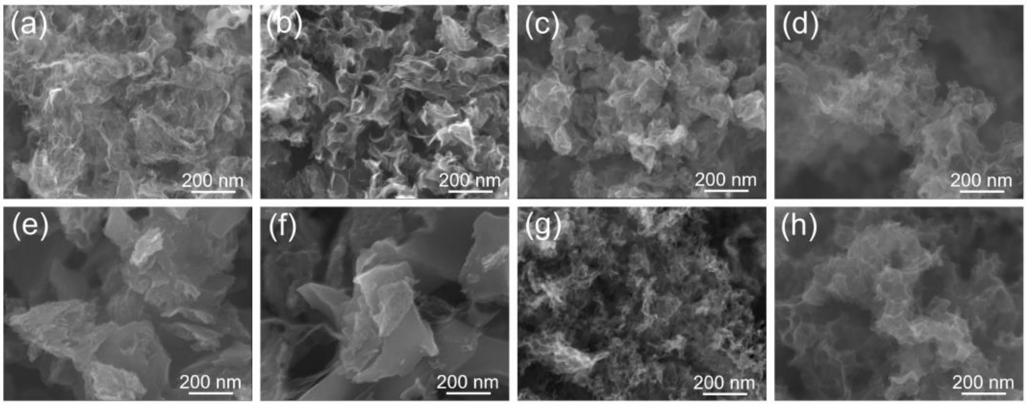

Figure S1. SEM images of (a) MCAN-0.5, (b) MCAN-2, (c) MCAN-3, (d) MCAN-0, (e) MAN-1, (f) CAN-1, (g) MCA-1 and (h) N-1.

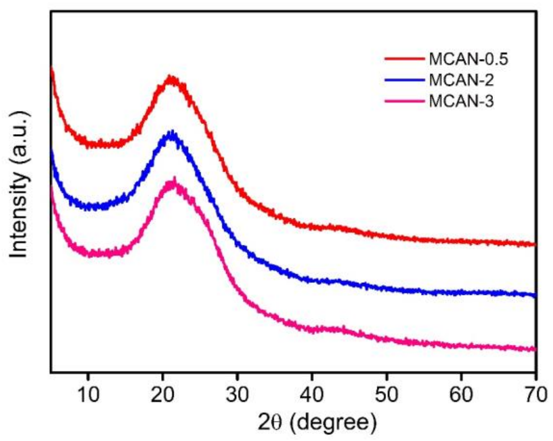

Figure S2. XRD patterns of MCAN-0.5, MCAN-2 and MCAN-3.

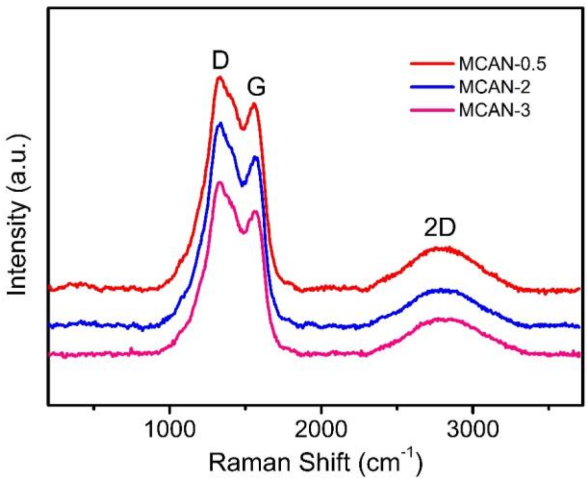

Figure S3. Raman spectra of MCAN-0.5, MCAN-2 and MCAN-3.

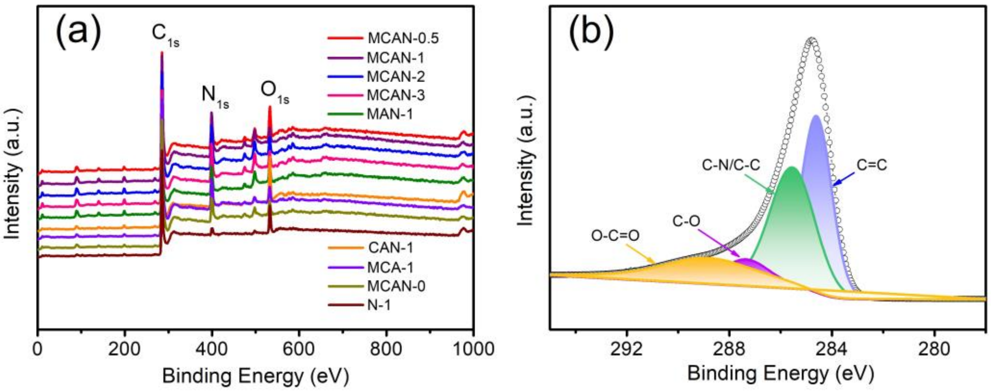

Figure S4. (a) Full-scan XPS spectra of all the samples and (b) the high-resolution XPS spectra of C 1s of MCAN-1.

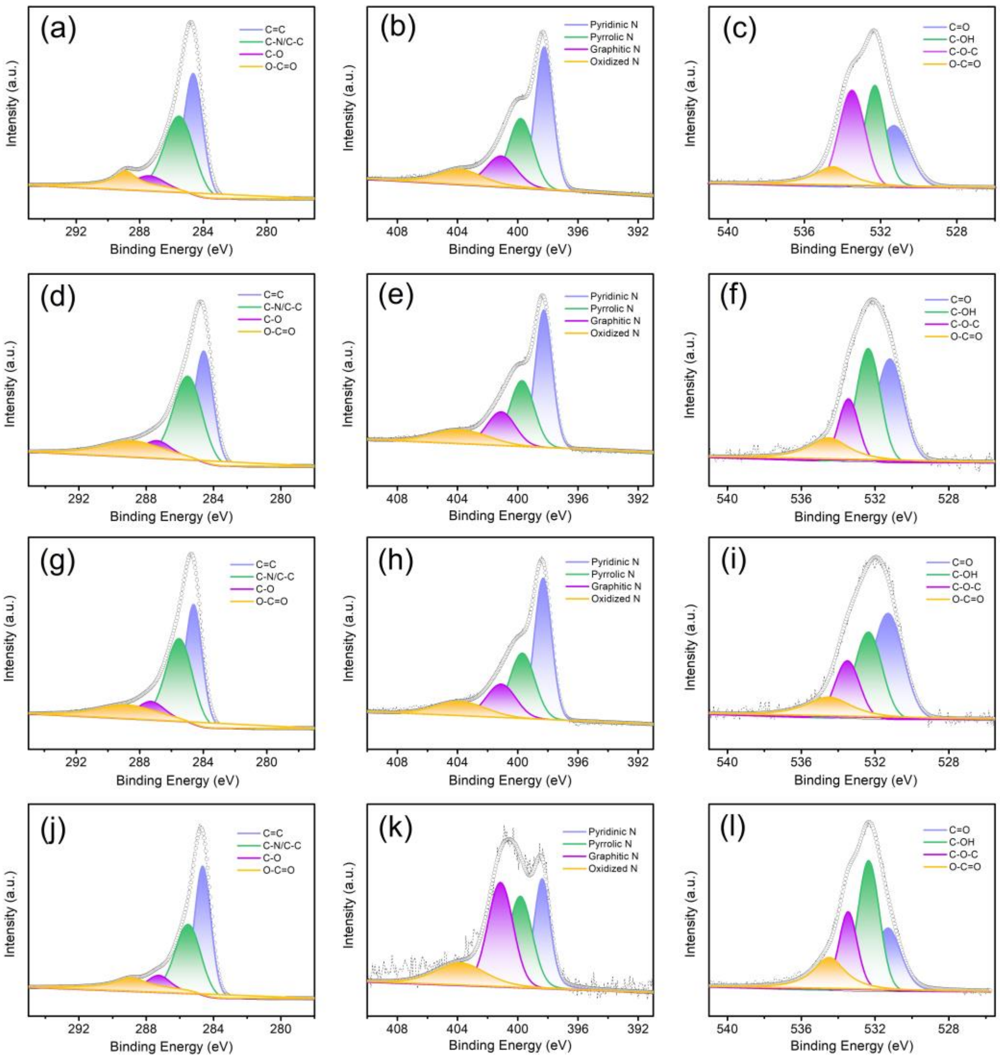

Figure S5. High-resolution XPS spectra of C 1s of (a) MCAN-0.5, (d) MCAN-2, (g) MCAN-3, (j) N-1. High-resolution XPS spectra of N 1s of (b) MCAN-0.5, (e) MCAN-2, (h) MCAN-3, (k) N-1. High-resolution XPS spectra of O 1s of (c) MCAN-0.5, (f) MCAN-2, (i) MCAN-3, (l) N-1.

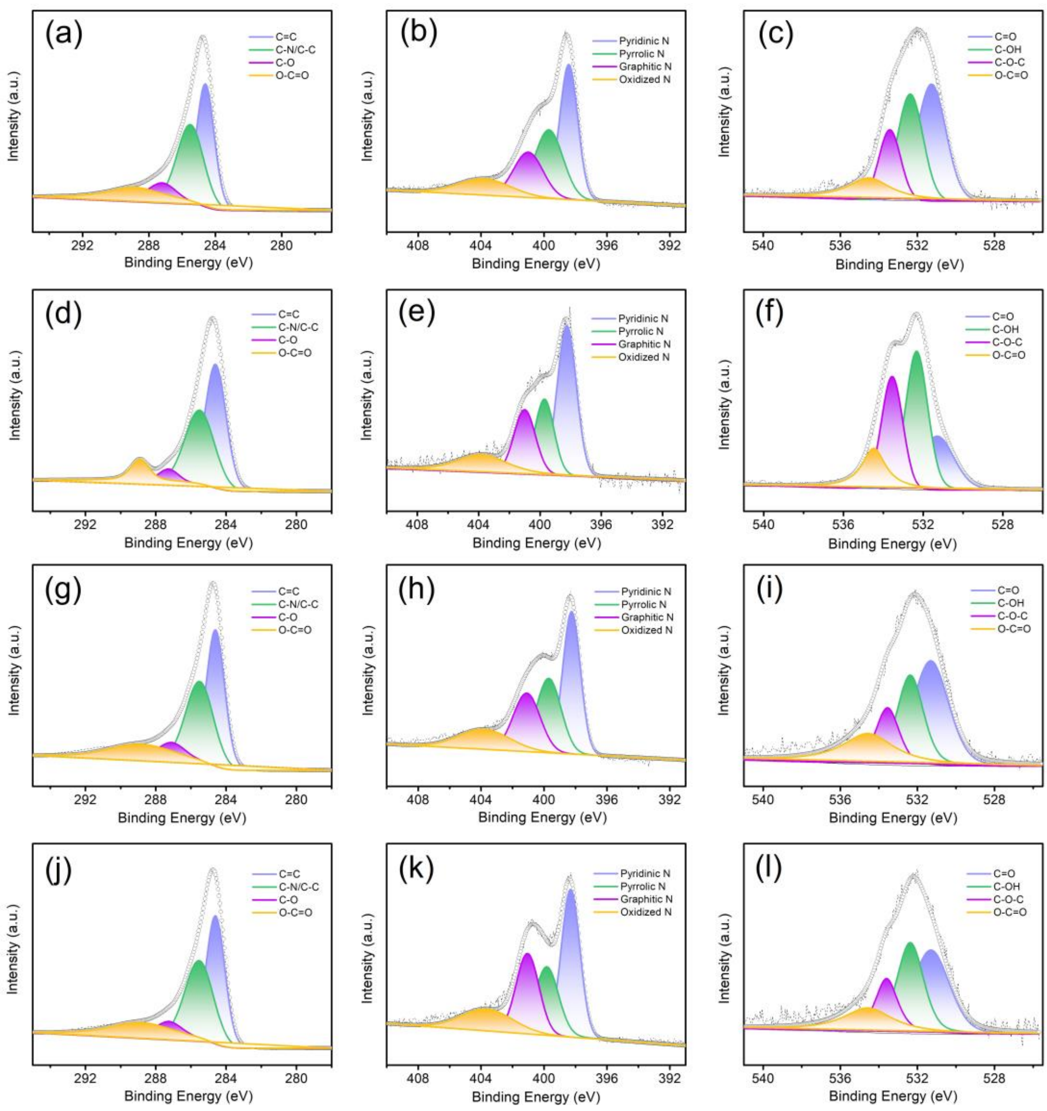

Figure S6. High-resolution XPS spectra of C 1s of (a) MAN-1, (d) CAN-1, (g) MCA-1, (j) MCAN-0. High-resolution XPS spectra of N 1s of (b) MAN-1, (e) CAN-1, (h) MCA-1, (k) MCAN-0. High-resolution XPS spectra of O 1s of (c) MAN-1, (f) CAN-1, (i) MCA-1, (l) MCAN-0.

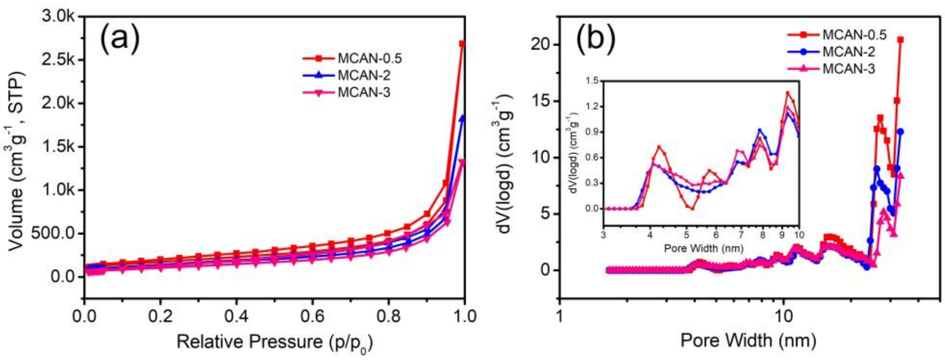

Figure S7. (a) N_2 adsorption/desorption isotherm and (b) pore size distribution (illustration: enlarged pore size distributions at about 3~10 nm) of MCAN-0.5, MCAN-2 and MCAN-3.

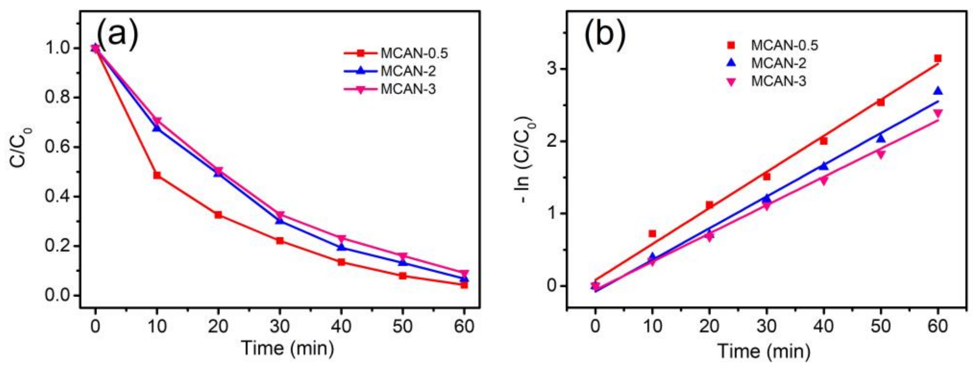

Figure S8. (a) EF degradation of BF by MCAN-0.5, MCAN-2 and MCAN-3 and (b) the corresponding variation of -ln(C/C_0).

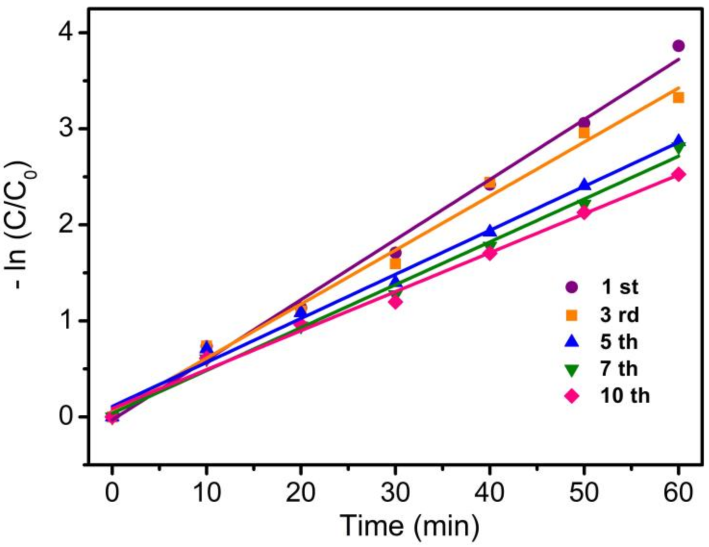

Figure S9. Variation of -ln(C/C_0) for BF degradation with MCAN-1 as cathode in 10 cycles.

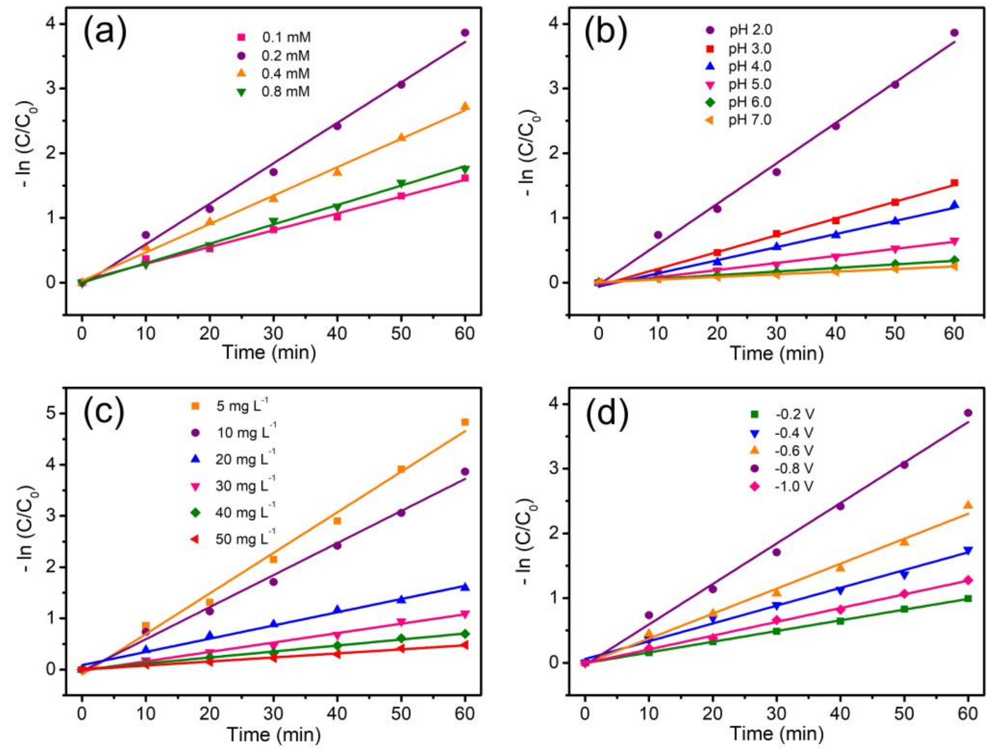

Figure S10. Variation of -ln(C/C_0) for BF degradation with MCAN-1 as cathode under different (a) Fe^{2+} contents, (b) pH values, (c) initial concentrations and (d) potentials.

Table S1. The rate constant k in 10 cycles.

Cycles	k [min^{-1}]	R^2
1 st	0.063	0.992
3 rd	0.046	0.991
5 th	0.046	0.991
7 th	0.045	0.991
10 th	0.041	0.990

Table S2. The rate constant k under different influencing parameters, *i.e.* cathode potential, Fe^{2+} content, initial concentration (C_0), and pH value.

potential [V]	Fe^{2+} content [mM]	C_0 [mg L^{-1}]	pH	k [min^{-1}]	R^2
-0.2	0.2	10	2	0.017	0.999
-0.4	0.2	10	2	0.028	0.990
-0.6	0.2	10	2	0.039	0.990
-1.0	0.2	10	2	0.021	0.997
-0.8	0.1	10	2	0.026	0.993
-0.8	0.4	10	2	0.043	0.995
-0.8	0.8	10	2	0.030	0.996
-0.8	0.2	5	2	0.079	0.990
-0.8	0.2	20	2	0.026	0.989
-0.8	0.2	30	2	0.018	0.990
-0.8	0.2	40	2	0.012	0.995
-0.8	0.2	50	2	0.008	0.992
-0.8	0.2	10	3	0.026	0.996
-0.8	0.2	10	4	0.020	0.992
-0.8	0.2	10	5	0.011	0.994
-0.8	0.2	10	6	0.005	0.991
-0.8	0.2	10	7	0.004	0.992
